# 17-Aminogeldanamycin selectively diminishes IRE1α-XBP1s pathway activity and cooperatively induces apoptosis with MEK1/2 and BRAF^V600E^ inhibitors in melanoma cells of different genetic subtypes

**DOI:** 10.1007/s10495-019-01542-y

**Published:** 2019-04-15

**Authors:** Aleksandra Mielczarek-Lewandowska, Malgorzata Sztiller-Sikorska, Marta Osrodek, Malgorzata Czyz, Mariusz L. Hartman

**Affiliations:** Department of Molecular Biology of Cancer, Medical University of Lodz, 6/8 Mazowiecka Street, 92-215 Lodz, Poland

**Keywords:** 17-aminogeldanamycin, Endoplasmic reticulum stress, HSP90 inhibitors, IRE-1α, Melanoma, Targeted therapy

## Abstract

Outcomes of melanoma patient treatment remain unsatisfactory despite accessibility of oncoprotein-targeting drugs and immunotherapy. Here, we reported that 17-aminogeldanamycin more potently activated caspase-3/7 in BRAF^V600E^ melanoma cells than geldanamycin, another inhibitor of heat shock protein 90 (HSP90). 17-aminogeldanamycin alleviated self-triggered compensatory increase in HSP70 mRNA level and induced endoplasmic reticulum (ER) stress, which was followed by selective diminution of cytoprotective IRE1α-XBP1s pathway activity of unfolded protein response (UPR), inhibition of ERK1/2 activity and induction of apoptosis. Concomitantly, ATF6/p50 level and expression of PERK-dependent genes, *CHOP* and *BIM*, remained unaltered. This might result from an inframe deletion in *EIF2AK3* leading to a PERK^L21del^ variant revealed by whole-exome sequencing in melanoma cell lines. 17-aminogeldanamycin exhibited similar activity in NRAS^Q61R^ melanoma cells that harbored a heterozygous inactivating variant of NAD(P)H:quinone oxidoreductase 1 (NQO1^P187S^). In addition, 17-aminogeldanamycin acted cooperatively with trametinib (an inhibitor of MEK1/2) and vemurafenib (an inhibitor of BRAF^V600E^) in induction of apoptosis in melanoma cell lines as evidenced by in-cell caspase-3/7 activation and PARP cleavage that occurred earlier compared with either drug used alone. As trametinib and vemurafenib did not significantly affect HSP70 and GRP78 transcript levels, cooperation of MEK/BRAF^V600E^ inhibitors and 17-aminogeldanamycin might result from a concurrent inhibition of the RAS/RAF/MEK/ERK cascade and IRE1α-dependent signaling, and cell-intrinsic ER homeostasis can determine the extent of the drug cooperation. Our study indicates that 17-aminogeldanamycin takes several advantages compared with other HSP90-targeting compounds, and can complement activity of BRAF/MEK inhibitors in melanoma cells of different genetic subtypes.

**Electronic supplementary material:**

The online version of this article (10.1007/s10495-019-01542-y) contains supplementary material, which is available to authorized users.

## Introduction

Genomic classification has been a gauge for clinical management of melanoma patients by using immunotherapy or targeted inhibitors of BRAF^V600^ and MEK1/2 [1]. However, lack of hot-spot *BRAF*, *RAS*, or *NF1* driver mutations in the triple wild-type subtype accounting for 6–20% of melanomas [2, 3], and variability of phenotype of patient-derived melanoma cell lines representing the same genetic subtype [4] enforce combining both genetic and phenotypic traits to achieve more accurately stratification of melanoma patients. In addition, phenotype-based approaches can limit the number of potential therapeutic targets by pointing to master regulators of cell identity as demonstrated by selection of either MEK or HSP90, whose inhibition substantially affected 75% of melanoma cell lines [5]. Heat shock protein 90 (HSP90) is a molecular chaperone involved in a proper folding and multiprotein complex assembly of a myriad of client proteins including several oncoproteins [6, 7], whereas a membrane-bound HSP90 in dying cells facilitates activation of the immune clearance [8]. *HSP90* is frequently overexpressed in cancer [6]. Accordingly, expression of *HSP90* substantially increases from nevi to melanoma resulting in high HSP90 level in more than 50% of melanoma tumors, and augments with advanced melanoma stage [9, 10]. In addition, also serum levels of HSP90 are higher in melanoma patients than in healthy controls, with median values 49.76 ng/ml versus 27.07 ng/ml, respectively [11]. More interestingly, it has been demonstrated that HSP90 isoform present in melanoma-derived exosomes contributes to creation of a pre-metastatic niche by ‘educating’ bone marrow progenitors [12].

HSP90 predominantly exerts its function via N-terminal ATPase domain, thus preventing from ATP binding largely interferes with HSP90 activity [13]. Regarding a pleiotropic role of this chaperone, inhibition of HSP90 is associated with an accumulation of improperly folded client proteins, which is followed by induction of endoplasmic reticulum (ER) stress and unfolded protein response (UPR) governed by glucose-regulated protein 78/binding immunoglobulin protein (GRP78/BiP). UPR engages three pathways initiated by the GRP78/BiP release of inositol-requiring enzyme 1 alpha (IRE1α), protein kinase R-like endoplasmic reticulum kinase (PERK) and activating transcription factor 6 (ATF6). These pathways either restore cell homeostasis or promote cell death in case of an excessive proteotoxic stress [14]. In preclinical melanoma studies, structurally different inhibitors of HSP90 produced ER stress [15], induced apoptosis and reduced tumorigenicity of vemurafenib-resistant cells [16, 17], circumvented mitochondria biogenesis [18] and mitigated immunosuppressing activity of melanoma cells [19]. Combining XL888 (Exelixis), a non-benzoquinone ATP-competitive inhibitor of HSP90, with targeted inhibitors of the RAS/RAF/MEK/ERK (MAPK) signaling pathway (XL888 + vemurafenib, and XL888 + vemurafenib + cobimetinib) is currently evaluated in phase I clinical trials in patients with unresectable melanoma (clinicaltrials.gov). In a dose escalation trial of XL888 and vemurafenib combination, 15 out of 20 patients (75%) responded to the treatment with a median overall survival of 34.6 months [20]. Resistance to a combination of XL888 and BRAF^V600^ inhibitor has been recently linked to a CDK2^high^/MITF^high^ phenotype of melanoma cells [21]. Concerning high protein levels of both MITF and CDK2 reported in five out of 12 melanoma cell lines [22] and the most significant correlation between MITF and CDK2 mRNA levels in melanoma tumor samples compared with other types of cancer [21], XL888 and BRAF^V600^ inhibitor combination is likely ineffective in a subset of patients. In the study by Azimi et al., it has been also demonstrated that the same melanoma cell line can exhibit a variable sensitivity to different HSP90 inhibitors [21]. It might result from dissimilar chemical structures of these compounds underlying execution of specific molecular effects as exemplified by BRAF^V600E^ degradation exhibited by benzoquinone inhibitors of HSP90 [23]. Therefore, further research on inhibitors structurally unrelated to XL888 is of interest.

Geldanamycin, a natural benzoquinone inhibitor of the N-terminal ATPase activity of HSP90, was first purified from *Streptomyces hydroscopicus*, and has been a prototype of a class of anti-cancer agents [24]. Geldanamycin-induced toxicity and low solubility have limited its clinical use [24]. Its derivatives, 17-substituted geldanamycin analogues are less hepatotoxic [25, 26]. 17-aminogeldanamycin is a metabolic product of cytochrome P450 3A4 (CYP3A4)-dependent conversion of 17-N-allylamino-17-demethoxygeldanamycin (tanespimycin) [27], and exhibits higher water solubility than a parental compound, and higher affinity to HSP90 than a number of other geldanamycin derivatives probably due to additional hydrogen bonds engaging an amine group [28]. It has been demonstrated that 17-aminogeldanamycin is a bioavailable compound upon oral administration [29, 30], and can reduce tumor growth and vessel density in xenografts of gastrointestinal stromal tumors (GIST) [31]. In our previous study on 120 natural agents, we have found that 17-aminogeldanamycin is more cytotoxic than geldanamycin, and both compounds are more potent against melanoma than leukemic cells [32]. In addition, 17-aminogeldanamycin significantly reduces c-MYC transcript level while not affecting the frequency of cells positive for ATP-binding cassette, sub-family B, member 5 (ABCB5) [32], which is a drug efflux transporter that mediates chemoresistance and marks melanoma-initiating cells [33]. These preliminary results prompted us to evaluate the activity of 17-aminogeldanamycin in melanoma cells more extensively.

## Materials and methods

### Cell culture

Melanoma cell lines were derived from tumors obtained during surgical interventions. The study was approved by Ethical Commission of Medical University of Lodz, and informed consent was obtained from all patients. Tumor fragments were washed, minced with scissors and incubated in HBSS (Sigma-Aldrich, St Louis, MO, USA) supplemented with 3 mM CaCl_2_ and 1 mg/ml collagenase IV for few hours at 37 °C. 10 μg/ml DNase I was added and cells were filtered through a 70 μm pore size filter. Cells were cultured in a complete medium (RPMI-1640 supplemented with 10% FBS) for 1 day to remove dead and non-adherent cells. Then, they were transferred to serum-free stem cell medium (SCM) consisting of DMEM/F12 low osmolality medium (Gibco, Paisley, UK), B-27 supplement (Gibco), 10 μg/ml insulin, 1 ng/ml heparin, 10 ng/ml bFGF, 20 ng/ml EGF (BD Biosciences, San Jose, CA, USA), 100 IU/ml penicillin and 100 μg/ml streptomycin [34–36]. Cell lines were named DMBC12, DMBC21, DMBC28, DMBC29 and DMBC22 (Department of Molecular Biology of Cancer, DMBC). DMBC12, DMBC21, DMBC28 and DMBC29 cells were assigned to the *BRAF* subtype as they harbored either a homozygous (DMBC12) or heterozygous (DMBC21, DMBC28 and DMBC29) BRAF^V600E^ variant, whereas DMBC22 cells harbored a homozygous Q61R substitution in NRAS [4]. For experiments, melanoma cells were seeded at final density, and drugs were added after 2.5 h.

### Drugs

Vemurafenib and trametinib were purchased from Selleck Chemicals LLC (Houston, TX, USA), geldanamycin from Sigma-Aldrich and 17-aminogeldanamycin from BOC Sciences (Shirley, NY, USA). Chemical formulas of geldanamycin and 17-aminogeldanamycin were prepared using ISIS/Draw (version 2.3). Drug stocks were prepared in DMSO. For experiments, drugs were dissolved in the culture medium to final concentrations as following: 5 μM vemurafenib (PLX), 50 nM trametinib (TRA), 0,1 and 0,4 μM geldanamycin (GEL) and 17-aminogeldanamycin (AG).

### Whole-exome sequencing

Whole-exome sequencing was performed as described previously [4]. Raw data are available at ArrayExpress and European Nucleotide Archive (ENA) under the numbers E-MTAB-6978 and ERP109743, respectively. Functional effects of amino acid substitutions were predicted in silico by the Polyphen-2 software (genetics.bwh.harvard.edu/pph2/index.shtml). The Polyphen-2-based predictions were classified as benign (scores 0.000–0.449), possibly damaging (scores 0.450–0.959) or probably damaging (scores 0.960–1.000).

### A time-lapse fluorescent microscopy

Melanoma cells were grown in 96-well plates (8 × 10^3^ cells/well) and treated with drugs at indicated concentrations and IncuCyte Caspase-3/7 Apoptosis Assay Reagent at 4 μM for 3 days. Activation of caspase-3/7 was monitored every 3 h by using a time-lapse fluorescence microscope system IncuCyte ZOOM (IncuCyte, Essen Bioscience). Data were analyzed using the IncuCyte Zoom original software. Percent of cells with active caspase-3/7 was calculated by dividing the percentages of confluence of apoptotic cells by the percentages of confluence of all cells at particular time points.

### Acid phosphatase activity assay

Acid phosphatase activity was assessed to determine a number of viable melanoma cells. Melanoma cells were grown for 0, 24, 48 and 72 h, then the plates were centrifuged and medium was replaced with assay buffer as described previously [34]. The absorbance values were measured using a microplate reader Infinite M200Pro (Tecan, Salzburg, Austria).

### Flow cytometry

Cells were incubated with drugs for 24 and 48 h, then collected, trypsinized and stained with Annexin V-FLUOS Staining Kit (Roche, Manheim, Germany) for 15 min. Flow cytometric data were acquired with FACSVerse (BD Biosciences), and analyzed using BD FACSuite.

### Cell lysate preparation and Western blotting

Melanoma cells were lysed in RIPA buffer containing 50 mmol/l Tris–HCl pH 8.0, 150 mmol/l NaCl, 1% TritonX-100, 0.5% sodium deoxycholate, 0.1% SDS supplemented with freshly added protease and phosphatase inhibitors (Sigma-Aldrich). Cell lysates were diluted in 2 × Laemmli buffer and protein samples (15 μg) were loaded on standard 7% SDS–polyacrylamide gel. After electrophoresis, the proteins were transferred onto Immobilon-P PVDF membrane (Millipore, Billerica, MA, USA) followed by incubation in a blocking solution: 5% nonfat milk in PBS-Tween 0.05% or 5% phospho-BLOCKER (Cell Biolabs, San Diego, CA, USA) in PBS-Tween 0.05%. Primary antibodies detecting PARP, ATF6, IRE1α, GRP78 and p53 were from Santa Cruz Biotechnology (Santa Cruz, CA, USA), p-IRE1α from Abcam (Cambridge, UK), β-actin from Sigma-Aldrich, p-ERK1/2 (Thr^202^/Tyr^204^) and ERK1/2 from Cell Signaling Technology (Danvers, MA, USA). Secondary HRP-conjugated anti-mouse or anti-rabbit antibodies (Santa Cruz Biotechnology) and Pierce ECL Western Blotting Substrate (Pierce, Rockford, IL, USA) were used to visualize proteins on the X-ray film (Foton-Bis, Bydgoszcz, Poland) or by using ChemiDoc Imaging System (Biorad). The quantification of the Western blotting data was performed by using ImageJ software.

### RNA isolation, cDNA synthesis and quantitative RT-PCR (qRT-PCR)

Total RNA isolation, cDNA synthesis and amplification procedures were extensively described elsewhere [32]. Primer sequences are shown in the Online Resource 1. To calculate the relative expression of target genes versus a reference gene *RPS17*, a mathematical model including an efficiency correction was used.

### Statistical analysis

Graphs are presented as mean ± SD. To calculate statistical significance of differences, Statistica v.13 software was used. Normality of a sample distribution was assessed by the Shapiro–Wilk test. The Levene’s test was used to determine equality of variances. The unpaired *t* test was used to compare two samples with normal distribution and equal variances. In case of different sample size or when n = 2, Mann–Whitney U test was used. To compare three samples with normal distribution and equal variances, ANOVA was used. The differences were considered significant if *p *≤ 0.05.

## Results

### 17-Aminogeldanamycin (AG) is more effective than geldanamycin (GEL) in caspase-3/7 activation in melanoma cells

**Fig. 1:**
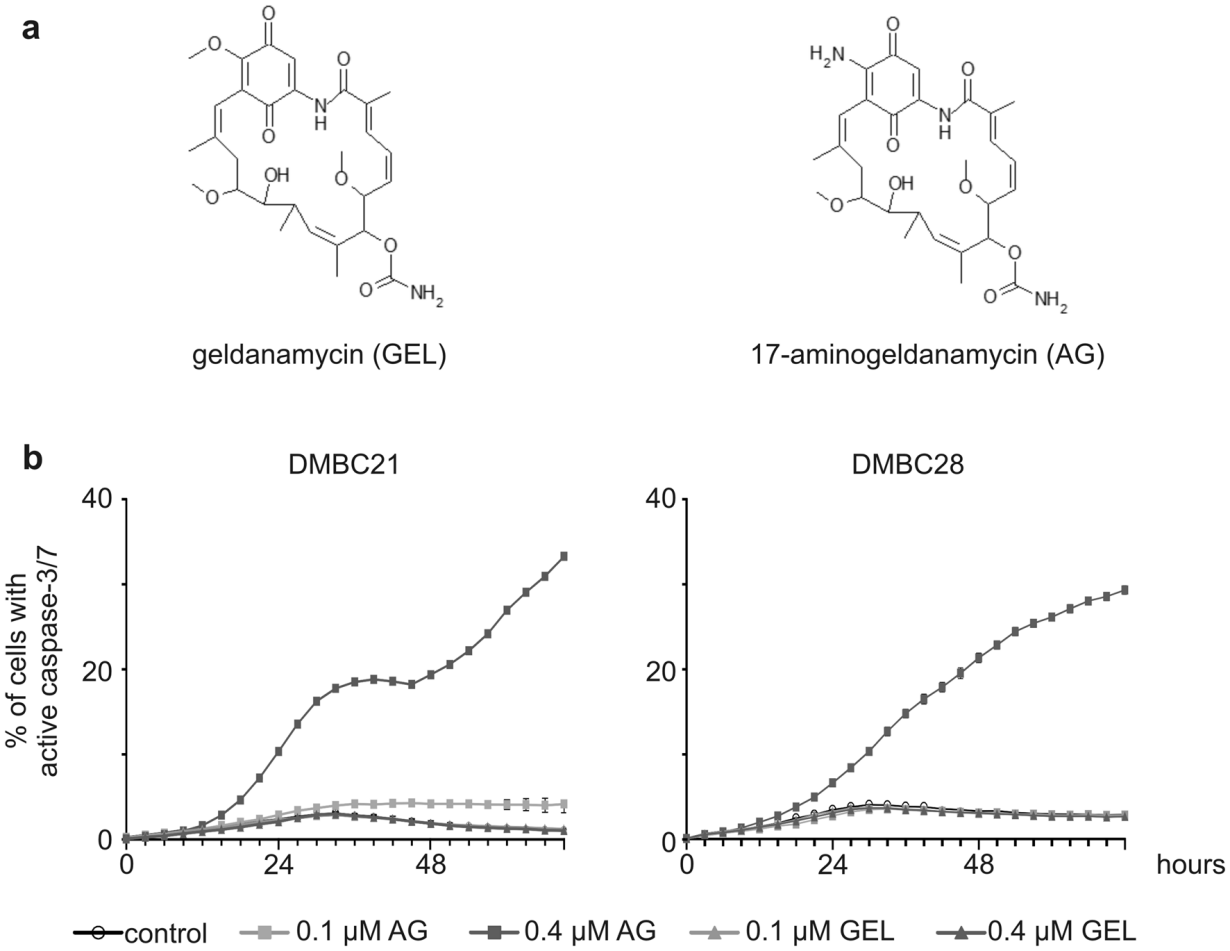
17-Aminogeldanamycin (AG) is more potent than geldanamycin (GEL) against BRAF^V600E^ melanoma cell lines **a** Structural formulas of GEL and AG. **b** Percentages of cells with active caspase-3/7 were assessed by time-lapse imaging system IncuCyte ZOOM in DMBC21 and DMBC28 cell lines incubated with either AG or GEL at indicated concentrations for 72 h

17-aminogeldanamycin (AG) is a geldanamycin (GEL) derivative in which a methoxy substituent attached to the C^17^ of the benzoquinone moiety is replaced by an amine group (Fig. 1a). First, we compared efficiency of GEL and AG in induction of apoptosis in two BRAF^V600E^ patient-derived melanoma cell lines, DMBC21 and DMBC28. Using a time-lapse fluorescence microscopy, we showed that 0.4 μM AG caused in-cell caspase-3/7 activation already after 24 h. This was followed by further increase in the percentages of cells with active caspase-3/7 up to 30–35% and cell detachment (Fig. 1b; Online Resource 3), whereas GEL at this concentration was ineffective.

### AG at 0.4 μM reduces viability and inhibits ERK1/2 activity in BRAF^V600E^ melanoma cells

**Fig. 2:**
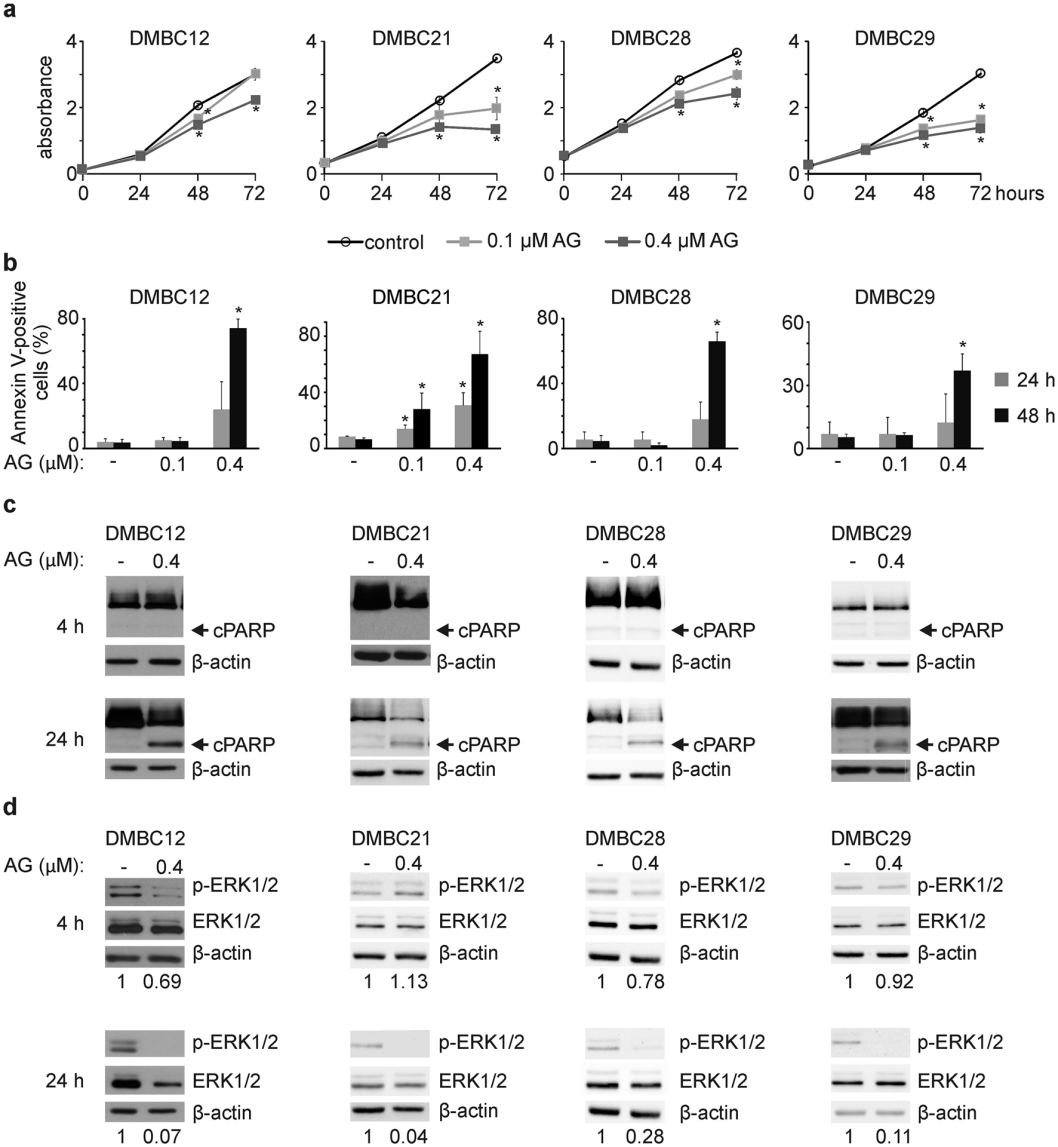
17-Aminogeldanamycin (AG) reduces viable cell number and inhibits activity of the MAPK signaling pathway **a** Melanoma cells were incubated with AG at 0.1 μM or 0.4 μM. Changes in viable cell number were assessed by acid phosphatase activity assay over the course of 72 h. Representative results are shown. *p ≤ 0.05 vs. control **b–c** Induction of apoptosis by AG is shown as percentages of Annexin V-positive cells after 24 and 48 h (*p ≤ 0.05 vs. control), and the level of cleaved PARP (cPARP) after 4 and 24 h. An equal loading was confirmed by β-actin. **d** Level of phosphorylated ERK1/2 (p-ERK1/2) was determined by Western Blotting after 4 and 24 h of cell incubation with 0.4 μM AG. β-actin was used as a loading control. p-ERK1/2 level was normalized to β-actin level, and shown below the blots

We used additional BRAF^V600E^ patient-derived cell lines to assess AG activity more extensively. AG reduced viable cell numbers after 72 h by 26, 62, 34 and 54% in DMBC12, DMBC21, DMBC28 and DMBC29 cell lines, respectively, as assessed by acid phosphatase activity (Fig. 2a). Double Annexin V/propidium iodide staining followed by flow cytometry revealed that 0.4 μM AG increased the frequency of Annexin V-positive cells in a time-dependent manner (Fig. 2b), whereas AG at 0.1 μM did not consistently induce apoptosis (Fig. 2b) indicating that AG at this concentration could rather exhibit cytostatic effect in melanoma cell lines (Fig. 2a). Apoptosis induced by 0.4 μM AG was also confirmed by detection of a caspase-mediated cleavage product of PARP already after 24 h (Fig. 2c), consistently with caspase-3/7 activation shown in DMBC21 and DMBC28 cell lines (Fig. 1b). In addition, AG-dependent diminution of the MAPK signaling pathway activity was observed (Fig. 2d). While this effect was almost undetectable after 4 h of exposure to drug, longer incubation markedly reduced ERK1/2 activity in both ERK1/2^high^ (DMBC12) and ERK1/2^low^ (DMBC21, DMBC28 and DMBC29) melanoma cell lines (Fig. 2d).

### AG transiently increases mRNA levels of HSP70 and GRP78

**Fig. 3:**
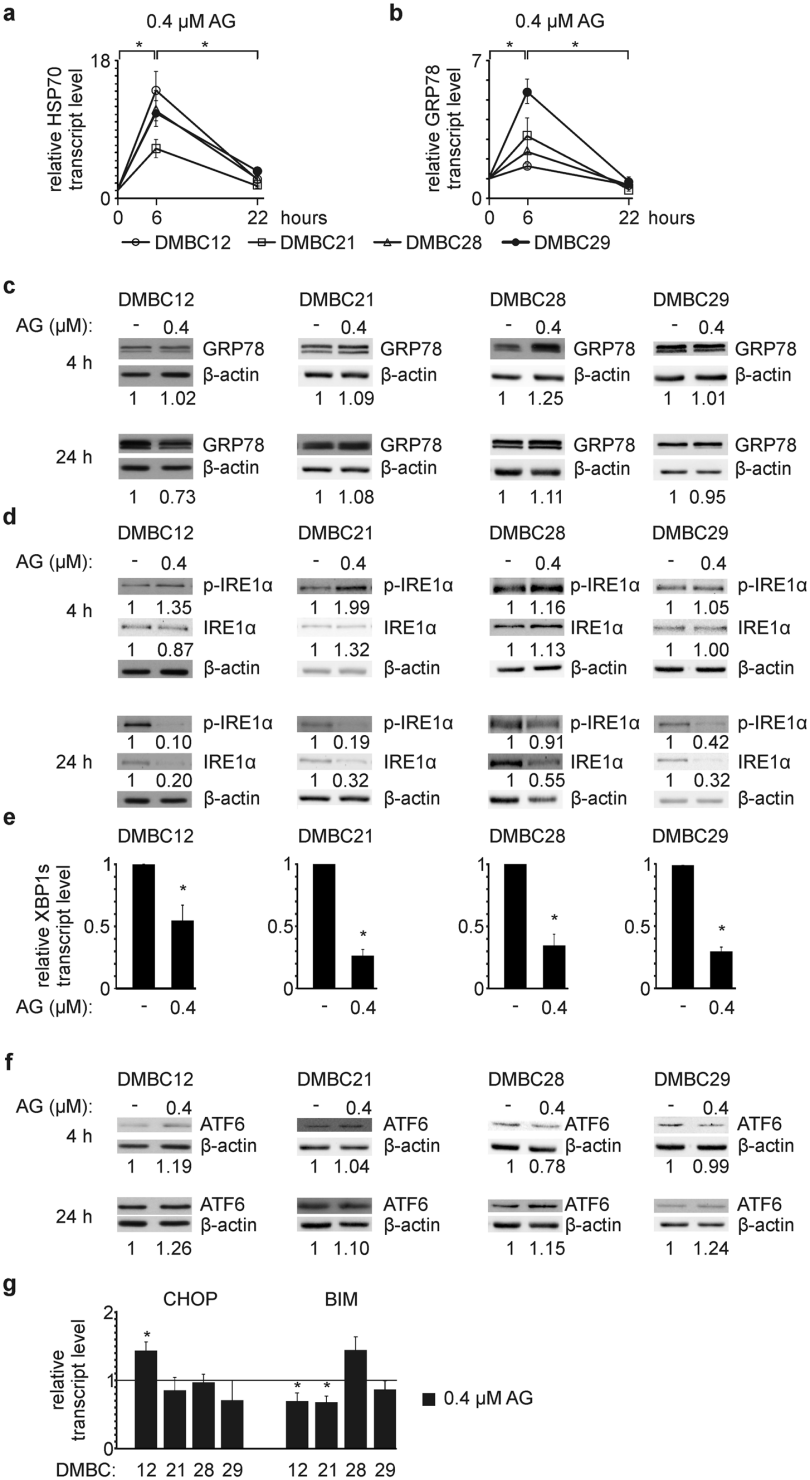
17-Aminogeldanamycin (AG) at 0.4 μM transiently increases mRNA levels of HSP70 and GRP78, and inhibits IRE1α-dependent pathway of UPR **a, b** HSP70 and GRP78 transcript levels were assessed by qRT-PCR after 6 and 22 h, and expressed relatively to the control. *p ≤ 0.05 **c** GRP78 protein level was determined by Western blotting after 4 and 24 h. An equal loading was confirmed by β-actin. Quantification of the protein level is shown below the blots. **d** Levels of active (phosphorylated IRE1α; p-IRE1α) and total IRE1α were determined after 4 and 24 h. β-actin was used as a loading control. Quantifications of p-IRE1α and IRE1α levels are shown below the blots. **e** XBP1 s transcript level after 22 h is shown relatively to the control. *p ≤ 0.05 **f** Activity of ATF6 was determined as the level of ATF6 cleavage product (p50) after 4 and 24 h. An equal loading was confirmed by β-actin. Quantification of the protein level is shown below the blots. **g** The transcript levels of CHOP and BIM were assessed by qRT-PCR after 22 h, and expressed relatively to the control. *p ≤ 0.05

Upregulation of *HSP70* expression compensates for attenuation of HSP90 activity by N-terminal inhibitors including geldanamycin and geldanamycin analogues [37]. AG at 0.4 μM significantly increased the transcript level of HSP70 after 6 h, which was a transient effect as HSP70 mRNA level decreased (*p *< 0.05) after additional 16 h of incubation with a drug (Fig. 3a). In addition, the transcript level of GRP78, a marker of ER stress induction, was significantly increased in all melanoma cell lines after 6 h, and reduced (*p *< 0.05) to the level of control after 22 h (Fig. 3b). Changes in *GRP78* expression were not associated with alterations in the level of corresponding protein assessed at two time intervals (Fig. 3c).

### AG selectively diminishes activity of IRE1α-dependent pathway of UPR

AG-mediated upregulation of *GRP78* expression suggested induction of ER stress. AG slightly increased IRE1α activity after 4 h (Fig. 3d), but levels of both total and phosphorylated IRE1α (p-IRE1α) were markedly reduced after additional 20 h of incubation with a drug (Fig. 3d). As a consequence, level of spliced XBP1 (XBP1s) mRNA was significantly diminished in all melanoma cell lines (Fig. 3e). Importantly, GEL at the same concentration did not affect the transcript level of XBP1s in DMBC21 and DMBC29 cells (Online Resource 4) that exhibited the largest decrease in XBP1s mRNA level in response to AG (Fig. 3e). Apart from its evident effect on the IRE1α-XBP1s pathway activity, 0.4 μM AG did not markedly affect level of a nuclear form of ATF6 (p50) (Fig. 3f), and did not significantly induce *CHOP* and *BIM* expression (Fig. 3g) encoding for executioners of PERK-dependent apoptosis. For that reason, we assessed p53/BIK-dependent route as an alternative pathway of apoptosis induced in response to prolonged ER stress [38]. AG at 0.4 μM did not substantially alter the protein level of p53, except for an increase in DMBC12 cells already after 4 h (Online Resource 5a). This was associated with an insignificant effect of AG on BIK transcript level in all melanoma cell lines (Online Resource 5b). To elucidate lack of apparent mechanism of ER stress-triggered apoptosis, we used whole-exome sequencing data [4] to determine the mutation status of genes encoding components of the UPR cascades. We found that PERK-dependent pathway might be affected by an inframe deletion in *EIF2AK3* leading to a PERK^L21del^ variant that was harbored in all melanoma cell lines (Online Resource 2), although genes encoding downstream components of the PERK signaling, ATF4 and eIF2α, either harbored alteration leading to a variant predicted as benign or were unaltered (Online Resource 2).

### AG cooperates with inhibitors of BRAF^V600E^ and MEK1/2 in induction of apoptosis

**Fig. 4:**
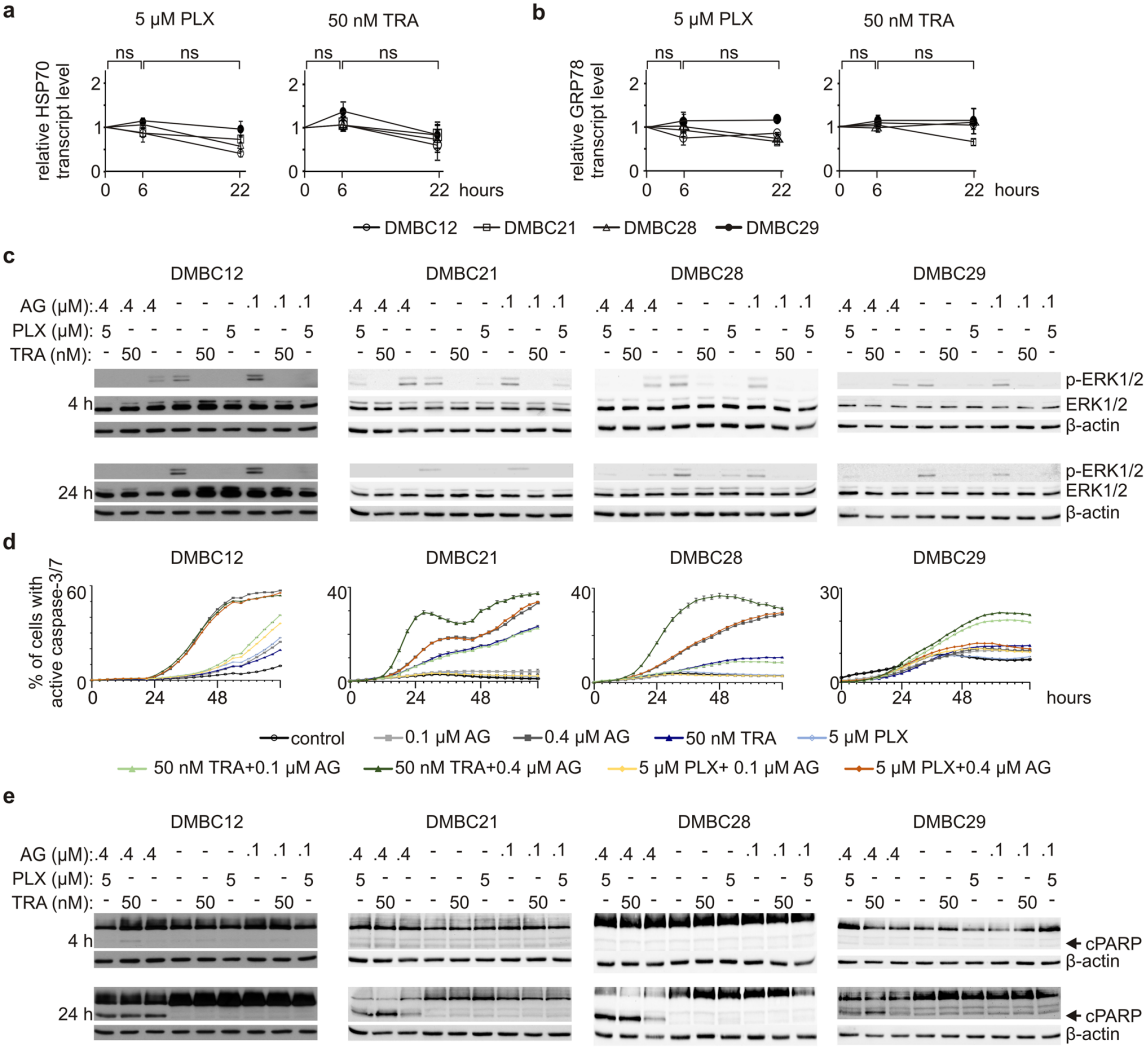
17-Aminogeldanamycin (AG) inhibits ERK1/2 activity and cooperates with trametinib (TRA) and vemurafenib (PLX) in induction of apoptosis in BRAF^V600E^ melanoma cell lines **a**, **b** HSP70 and GRP78 transcript levels were assessed by qRT-PCR after 6 and 22 h of cell incubation with either 5 μM PLX or 50 nM TRA, and expressed relatively to the control. *ns* not significant. **c**–**e** Melanoma cell lines were incubated with AG, PLX and TRA used either alone or in combinations at indicated concentrations. **c** Level of phosphorylated ERK1/2 (p-ERK1/2) was assessed by Western blotting after 4 and 24 h. An equal loading was confirmed by β-actin. **d** Percentages of cells with active caspase-3/7 were assessed by time-lapse imaging system IncuCyte ZOOM. **e** Level of cleaved PARP (cPARP) was determined after 4 and 24 h. An equal loading was confirmed by β-actin. Quantification of cPARP level after 24 h of cell incubation with drugs is shown in the Online Resource 7a

HSP90 is a chaperone protein for several oncoproteins that contribute to melanoma cell response to inhibitors of the MAPK signaling pathway, vemurafenib (PLX; an inhibitor of BRAF^V600E^) and trametinib (TRA; an inhibitor of MEK1/2). Therefore, we investigated whether a combination of AG and PLX or TRA could cooperatively induce apoptosis. In the following experiments, we used also 0.1 μM AG. AG already at this low concentration significantly increased HSP70 transcript level after 6 h, which was followed by a reduction (*p *< 0.05) after additional 16 h of incubation in all melanoma cell lines (Online Resource 6a). It also significantly augmented GRP78 mRNA level after 6 h in DMBC29 cells (Online Resource 6b) while not triggering apoptosis when used alone (Figs. 1b and 2b). PLX at 5 μM and TRA at 50 nM did not induce significant changes in the transcript levels of HSP70 (Fig. 4a) and GRP78 (Fig. 4b) in all BRAF^V600E^ cell lines. As expected, PLX and TRA inhibited ERK1/2 activity already after 4 h (Fig. 4c). Similar effect was exerted by 0.4 μM AG after 24 h (Figs. 2d, 4c), whereas AG at 0.1 μM poorly affected level of phospho-ERK1/2 (Fig. 4c). In drug combinations, the effect of PLX and TRA was dominant, and AG did not interfere with PLX- and TRA-mediated attenuation of ERK1/2 activity (Fig. 4c). Real-time monitoring of cells incubated with a combination of 50 nM TRA and 0.4 μM AG revealed that activation of caspase-3/7 occurred earlier and in a larger number of cells compared with cells incubated with either drug used alone (Fig. 4d). For example, the percentage of cells with active caspase-3/7 was around or < 10% upon incubation with either 50 nM TRA or 0.4 μM AG, but reached almost 20% and 30% when a drug combination was used in DMBC21 and DMBC28 cells, respectively (Fig. 4d). This cooperatively induced apoptosis was observed in DMBC21, DMBC28 and DMBC29 cell lines, but not in DMBC12 cells (Fig. 4d), which was consistently reflected in the level of cleaved PARP after 24 h (Fig. 4e; Online Resource 7a). Combined effect of 5 μM PLX and 0.4 μM AG was also observed as induction of PARP cleavage, however, it was less pronounced than effect of the TRA + AG combination in DMBC21 and DMBC29 cell lines, and not observed in DMBC12 cells (Fig. 4e; Online Resource 7a). We also determined the level of IRE1α and XBP1s in melanoma cells exposed to PLX and TRA, and their combinations with AG. We found that PLX and TRA inhibited IRE1α activity in DMBC12 cells after 24 h, but it was associated with insignificant alteration in the transcript level of XBP1s (Online Resource 7b). PLX and TRA also slightly diminished levels of phospho-IRE1α and XBP1s in DMBC21 cells. In combination with AG, PLX/TRA did not cooperatively reduce the XBP1s mRNA level in any melanoma cell line (Online Resource 7b).

### AG is also effective against NRAS^Q61R^ melanoma cells

**Fig. 5:**
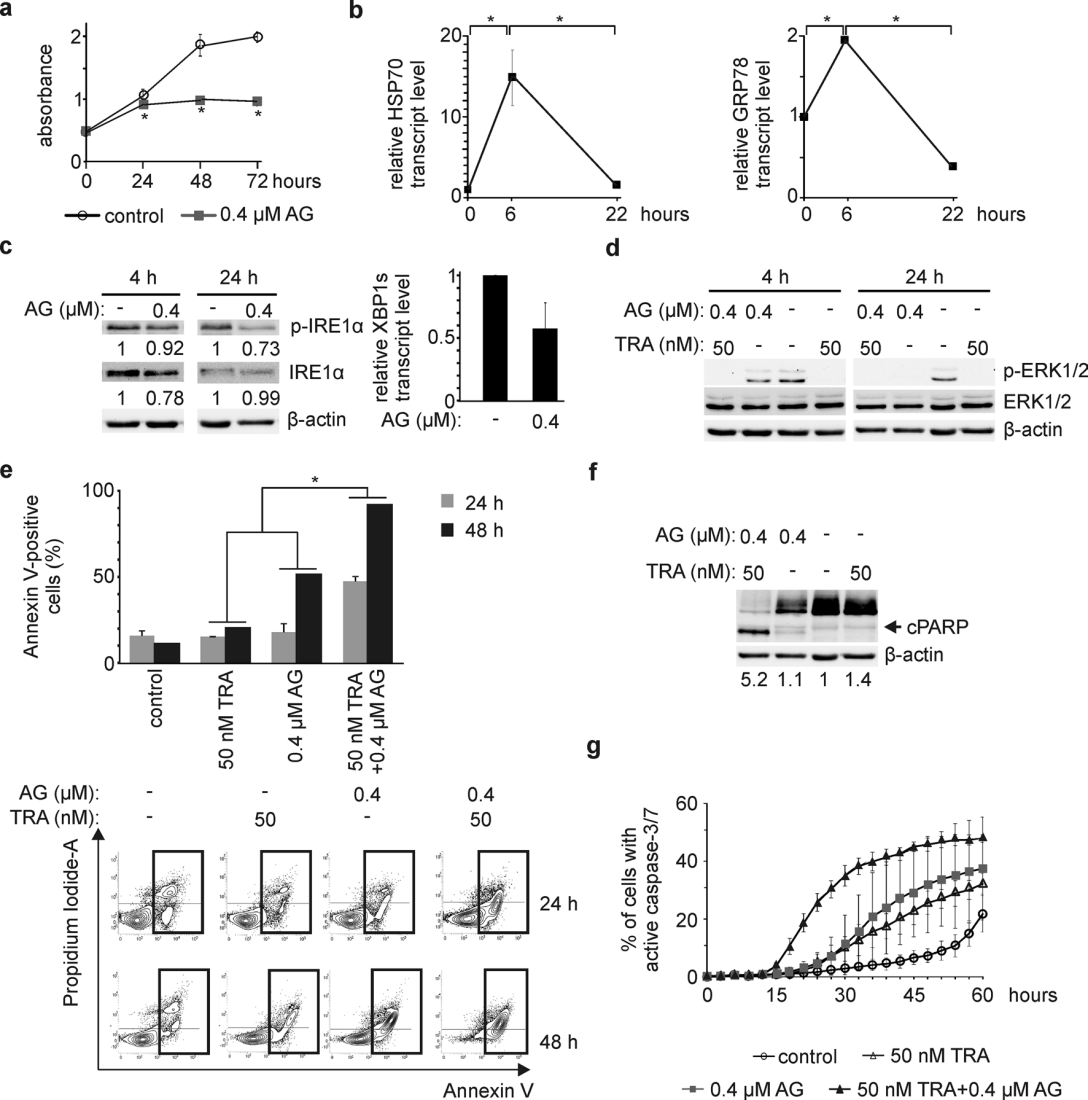
Cellular and molecular effects of 17-aminogeldanamycin (AG) activity used either alone or in combination with trametinib (TRA) in NRAS^Q61R^ melanoma cells **a** Changes in a viable cell number were assessed by acid phosphatase assay. Representative results are shown. *p ≤ 0.05 versus control **b** HSP70 and GRP78 transcript levels were assessed by qRT-PCR in cells incubated with 0.4 μM AG for 6 and 22 h, and expressed relatively to the control. *p ≤ 0.05 **c** Level of phospho-IRE1α (p-IRE1α) was determined after 4 and 24 h. β-actin was used as a loading control. Quantifications of p-IRE1α and IRE1α levels are shown below the blots. XBP1s transcript level was assessed by qRT-PCR after 22 h, and expressed relatively to control. **d**–**g** DMBC22 cells were incubated with 0.4 μM AG and 50 nM TRA used either alone or in combination. **d** Level of phosphorylated ERK1/2 (p-ERK1/2) was assessed by Western blotting, and β-actin was used as a loading control. **e** Percentages of Annexin V-positive cells after 24 and 48 h were determined by flow cytometry. Representative contour plots are shown. *p ≤ 0.05 drug combination vs. either drug used alone **f** Cleaved PARP (cPARP) was immunoblotted after 24 h. An equal loading was confirmed by β-actin. Quantification of cPARP level is shown below the blots. **g** Percentages of cells with active caspase-3/7 were assessed by time-lapse microscopy

We also employed DMBC22 cell line that was previously assigned to the *NRAS* subtype of melanoma as it harbored a homozygous NRAS^Q61R^ variant and a wild-type *BRAF* [4]. AG at 0.4 μM markedly reduced viability in DMBC22 cell line by 52% after 72 h as evidenced by changes in acid phosphatase activity (Fig. 5a), and significantly increased transcript levels of HSP70 and GRP78 after 6 h, which was followed by a decrease (*p *< 0.05) to the level of control after 22 h (Fig. 5b). In addition, 0.4 μM AG reduced the levels of phospho-IRE1α and XBP1s transcript (Fig. 5c). To elucidate whether drug cooperation reported in most of BRAF^V600E^ cell lines was also executed in NRAS^Q61R^ melanoma cells, DMBC22 cells were incubated with 50 nM TRA and 0.4 μM AG, used either alone or in combination. While TRA inhibited ERK1/2 activity already after 4 h, AG reduced level of phospho-ERK1/2 level after 24 h of incubation (Fig. 5d). Combination of TRA and AG increased the frequency of Annexin V-positive cells in a time-dependent manner (Fig. 5e) and augmented the level of cleaved PARP after 24 h (Fig. 5f) to larger extents than those observed for either drug used alone. Importantly, the occurrence of AG-induced apoptosis in DMBC22 cell line was slightly delayed compared to BRAF^V600E^ cells as consistently reported at the cellular and molecular levels (Fig. 5e, f vs. Fig. 2b, c). Notably, this was clearly visible in real-time measurement of the percentages of cells with active caspase-3/7 (Fig. 5g vs. Fig. 4d). Moreover, AG and TRA used in combination induced caspase-3/7 much earlier than either drug alone (Fig. 5g). For example, the percentage of cells with active caspase-3/7 was ~ 5% when cells were incubated with either 50 nM TRA or 0.4 μM AG, but reached ~ 25% when DMBC22 cells were exposed to a combination of drugs (Fig. 5g). In addition, p53-BIK axis was unlikely involved in the induction of apoptosis (Online Resource 5a, b), whereas PERK pathway-dependent upregulation of *CHOP* and *BIM* expression was not detected (data not shown) possibly due to a homozygous PERK^L21del^ variant harbored in DMBC22 cells similarly to BRAF^V600E^ cell lines (Online Resource 2).

## Discussion

In the present study, we have demonstrated that 17-aminogeldanamycin perturbates ER homeostasis, predominantly by interfering with activity of IRE1α-dependent pathway, and induces apoptosis in melanoma cells of the BRAF^V600E^ and NRAS^Q61R^ subtypes. 17-aminogeldanamycin takes several advantages over geldanamycin and other geldanamycin derivatives as supported by previously published and present results showing that 17-aminogeldanamycin (i) is more potent than geldanamycin against melanoma cells; (ii) exerts anti-melanoma activity at lower concentrations compared with IC_50_ values for other geldanamycin analogues assessed in different human cancer cell lines [26], and (iii) attenuates self-triggered increase in HSP70 transcript level.

### 17-Aminogeldanamycin alleviates self-triggered upregulation of HSP70 expression

It has been demonstrated that N-terminal inhibitors of HSP90 activate heat shock factor-1 (HSF-1) to upregulate expression of other chaperones including HSP70 and HSP27 [37, 39, 40]. This cell response contributes to development of resistance to HSP90 inhibitors [41, 42] as compensatory chaperones can remain at increased levels in cells incubated with geldanamycin, its analogues [43–45] and XL888, a geldanamycin-unrelated inhibitor of HSP90 [46]. Here, we have shown that 17-aminogeldanamycin upregulates *HSP70* expression, which returns to baseline mRNA level in both BRAF^V600E^ and NRAS^Q61R^ melanoma cell lines. It is consistent with other results showing insignificant alterations in the protein level of HSP70 in GIST cell lysates after 17-aminogeldanamycin administration [31]. This suggests that attenuation of self-induced upregulation of *HSP70* expression is rather drug-specific than cell type-dependent, and might be crucial for a high activity of 17-aminogeldanamycin in melanoma cells.

### 17-Aminogeldanamycin selectively inhibits cytoprotective IRE1α-XBP1s signaling pathway of UPR

Upregulation of HSP70 expression following inhibition of HSP90 compensates for HSP90 function and deters from activation of prolonged ER stress and UPR, and induction of apoptosis emerging from an increased burden of misfolded proteins [47]. Disturbances in the execution of UPR support adaptation of cancer cells to proteotoxic stress [48]. In melanoma, UPR and other stress-attenuating signaling pathways are hyperactivated during tumor progression and cell response to therapy [49, 50]. They engage different anti-apoptotic proteins [51] and microphthalmia-associated transcription factor (MITF) [52]. Increasing evidence suggests that targeting the ER stress and UPR programs can be a promising anti-melanoma strategy [53, 54], also against resistant cells due to the contribution of GRP78/BiP to ERK1/2 reactivation [55]. In the present study, we have found that 17-aminogeldanamycin induces ER stress as evidenced by a transient increase in GRP78 transcript level and a slight activation of IRE1α already after 4 h. IRE1α autophosphorylation followed by its dimerization leads to the excision of a 26-nt intron from XBP1 transcript and a generation of XBP1s mRNA translated into XBP1, a transcription factor that regulates expression of ER stress-attenuating genes [56, 57]. XBP1 has been implicated in the regulation of melanoma cell proliferation by activating inteleukin-6/STAT3 pathway [58] in addition to its critical role in melanoma cell survival during ER stress [59]. As both IRE1α and ATF6 can upregulate expression of *GRP78* in melanoma [60], the contribution of IRE1α to the increase in GRP78 mRNA level is more likely because ATF6 activity remained unaltered following 17-aminogeldanamycin treatment. More importantly, while IRE1α activation could be an acute cytoprotective response of melanoma cells to 17-aminogeldanamycin, prolonged incubation with this compound led to a substantial diminution of both activity and protein level of IRE1α, probably due to a chaperoning role of HSP90 on IRE1α [61]. This effect can be specific for particular HSP90 inhibitors because tanespimycin inhibited XBP1s generation without abrogating IRE1α protein level even when used in higher concentration (1 µM) [62] than 0.4 µM 17-aminogeldanamycin used in our study. Therefore, 17-aminogeldanamycin is capable to reduce IRE1α activity and also, by leading to IRE1α degradation, to prevent from undesired activation of cytoprotective IRE1α signaling, which might be valuable considering 17-aminogeldanamycin as a part of combined treatment.

### 17-Aminogeldanamycin induces apoptosis in melanoma cells harboring PERK^L21del^ and NQO1^P187S^ variants

ER stress-induced apoptosis has been commonly attributed to PERK signaling-dependent upregulation of *CHOP* and *BIM* expression [14], although CHOP-deficient cells also undergo apoptosis in response to ER stress inducers [63]. Alternatively, p53-dependent suppression of GRP78 mRNA translation and induction of *BIK* expression can induce apoptosis [38]. In the present study, we have demonstrated that apoptosis induced by 17-aminogeldanamycin unlikely depends on any of these pathways. PERK-dependent signaling might be affected due to an inframe deletion in *EIF2AK3* leading to a PERK^L21del^ variant that was found in all melanoma cell lines. A PERK^L21del^ variant has been already reported in one tumor sample excised from a patient before treatment with vemurafenib [64]. It needs to be elucidated how deletion of Leu^21^ affects PERK structure and activity. On the other hand, an increase in p53 level was found exclusively in one cell line and this was not associated with either decrease in GRP78 protein level (Fig. 3c) or upregulation of *BIK* expression (Online Resource 5), despite transcriptionally active p53 in all melanoma cell lines [4]. Thus, while 17-aminogeldanamycin apparently diminishes activity of cytoprotective IRE1α-XBP1s axis and induces apoptosis in melanoma cells, specific mediators of cell death need to be determined.

Cell sensitivity to geldanamycin derivatives correlates with expression of NAD(P)H:quinone oxidoreductase 1 (*NQO1*) [65], and NQO1-mediated quinone-to-hydroquinone conversion of geldanamycin and its analogues enhances activity of these compounds because of increased hydrogen bonding of hydroquinone derivatives [66]. It has also been shown that a P187S variant of NQO1 has a reduced activity compared with wild-type *NQO1* [67], but genetic alterations affecting a His^80^ residue in NQO1 can compensate for P187S substitution [68]. In our study, a heterozygous NQO1^P187S^ variant (rs1800566) was harbored exclusively in DMBC22 cells. As DMBC22 cells lack additional alterations in *NQO1*, NQO1^P187S^ variant might contribute to a delayed pro-apoptotic response of DMBC22 cells to 17-aminogeldanamycin compared with the response of cell lines harboring wild-type *NQO1*. However, it has been demonstrated that the affinity of 17-aminogeldanamycin to purified HSP90 is not considerably enhanced upon reduction to the hydroquinone [29], and cell response to 17-aminogeldanamycin is not associated with NQO1 protein level [31]. It suggests that activity of 17-aminogeldanamycin is at least partially independent of NQO1 that can be advantageous because a loss of *NQO1* expression and acquisition of an inactive NQO1^P187S^ variant have been demonstrated as causative factors in development of resistance to tanespimycin in melanoma and glioblastoma cells [69].

### 17-Aminogeldanamycin and inhibitors of the RAS/RAF/MEK signaling cascade cooperatively induce apoptosis

**Fig. 6:**
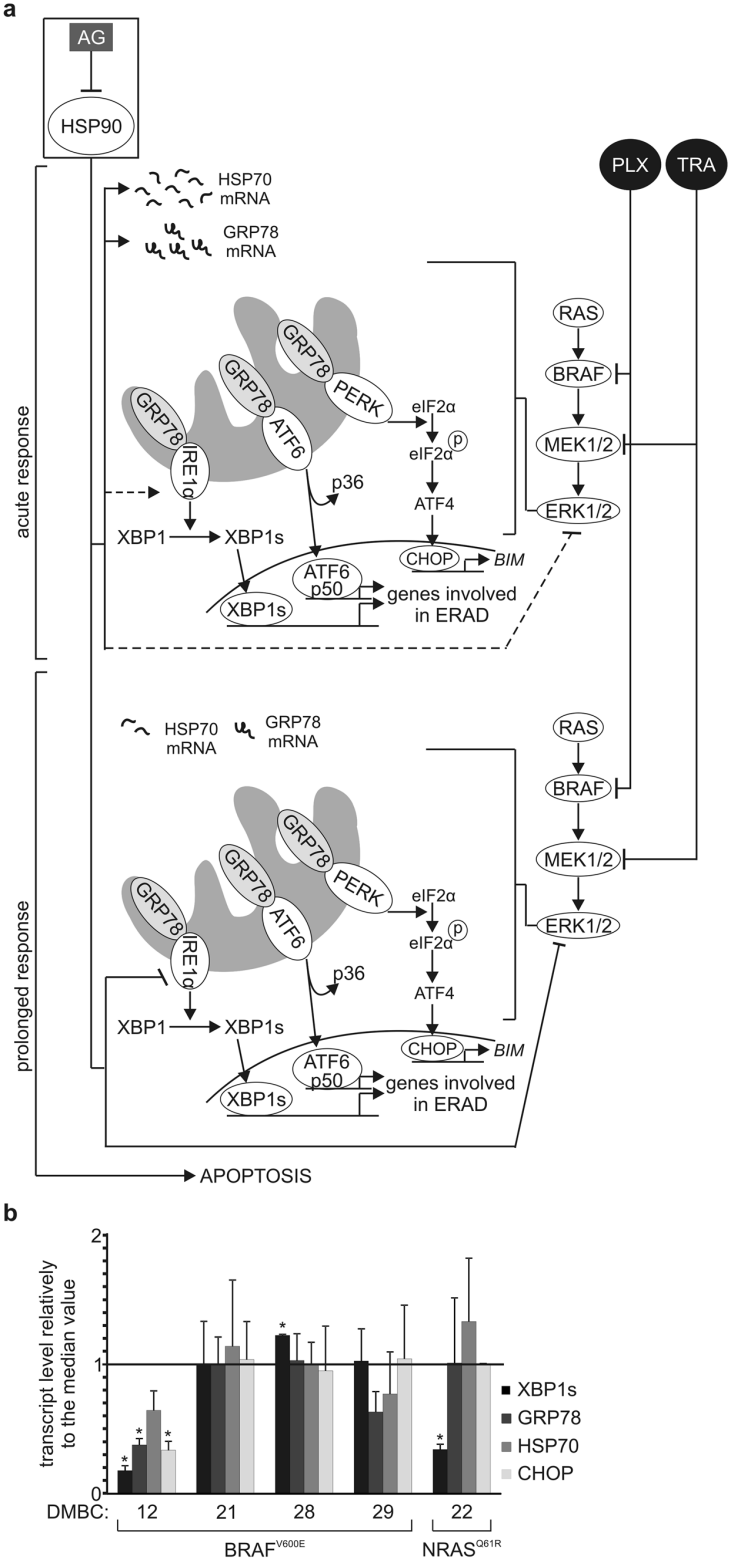
Cooperation of 17-aminogeldanamycin (AG) and trametinib (TRA) or vemurafenib (PLX) **a** A schematic summary of molecular effects of TRA, and PLX activity, and 17-aminogeldanamycin-mediated HSP90 inhibition in melanoma cells. Alterations accompanying acute (4–6 h) and prolonged (22–24 h) response are shown. See discussion for additional comments. ERAD, endoplasmic reticulum-associated protein degradation **b** Transcript levels of XBP1 s, GRP78, HSP70 and CHOP were determined by qRT-PCR in drug-naïve BRAF^V600E^ and NRAS^Q61R^ melanoma cell lines, and expressed relatively to the median value in all five cell lines. *p ≤ 0.05 versus median value

A tolerable side effect profile has been recently shown for a combination of HSP90 and BRAF^V600E^ inhibitors [20]. Clinical trials NCT01657591 and NCT02721459 on co-treatment of melanoma patients with XL888 and inhibitors of the MAPK signaling pathway are ongoing (clinicaltrials.gov). The rationale for combining these drugs is that several proteins, which are linked to melanoma cell response and resistance to BRAF^V600^/MEK-targeting agents, are HSP90 clients including ARAF, CRAF, BRAF^V600E^, CDK4, COT, IGF1R, PDGFR-β and AKT [16, 70, 71]. In addition, it has been demonstrated that oncogenic MAPK signaling increases intracellular protein load and maintains cytoprotective autophagy at elevated level [59, 72], and sustains IRE1α and ATF6 at activated states to adapt melanoma cells to a chronic ER stress [60]. Indeed, attenuation of the MAPK pathway activity sensitizes melanoma cells to ER stress-inducing drugs [73]. Moreover, it even directly causes the release of Ca^2+^ from endoplasmic reticulum [74] and enhances interaction between mutant BRAF and GRP78 to induce PERK-dependent apoptosis [75]. In our study, vemurafenib and trametinib did not upregulate *GRP78* expression, but affected IRE1α-XBP1s signaling in certain melanoma cell lines. Therefore, we propose a putative model of cooperation between 17-aminogeldanamycin and trametinib or vemurafenib (Fig. 6a). According to this model, 17-aminogeldanamycin exerts dual time-dependent activity. By inhibiting HSP90, 17-aminogeldanamycin triggers accumulation of unfolded proteins thereby rapidly inducing ER stress as evidenced by an increase in GRP78 and HSP70 transcript levels and a slight activation of IRE1α. It is followed, however, by a decay of IRE1α protein, which results in the attenuation of cytoprotective IRE1α-XBP1s axis, in addition to a diminution of the transcript levels of HSP70 and GRP78 as well as activity of ERK1/2. ERK1/2 maintains basal ER homeostasis in melanoma cells. In comparison to 17-aminogeldanamycin, trametinib and vemurafenib rapidly attenuate ERK1/2 activity, which might be crucial for potentiating effects triggered by 17-aminogeldanamycin. Consequently, apoptosis is induced earlier and in a larger number of cells compared with either drug used alone, but not in all cell lines. We have found that the transcript levels of XBP1s, GRP78, HSP70 and CHOP were the lowest in drug-naïve DMBC12 cells compared with other BRAF^V600E^ and NRAS^Q61R^ melanoma cell lines (Fig. 6b). Already low adaptation to ER disturbances might determine lack of cooperation between 17-aminogeldanamycin and targeted therapeutics in DMBC12 cells. Therefore, our study suggests that cooperatively induced apoptosis might result from a concurrent inhibition of both, the MAPK signaling and cytoprotective IRE1α-XBP1s axis, and cell-intrinsic ER homeostasis can narrow the extent of drug cooperation.

## Conclusion

17-Aminogeldanamycin is a more potent HSP90 inhibitor than geldanamycin against melanoma cells, and exerts previously unidentified activities including diminution of self-triggered upregulation of *HSP70* expression and selective inhibition of cytoprotective IRE1α-XBP1s axis of unfolded protein response. In addition, 17-aminogeldanamycin cooperates with inhibitors of the MAPK signaling pathway in apoptosis induction that might be exploited in BRAF^V600E^ and NRAS^Q61R^ melanomas.

## Electronic supplementary material

Below is the link to the electronic supplementary material.

Supplementary material 1 (DOC 6160 kb)

## Abbreviations

AG: 17-Aminogeldanamycin
ATF6: Activating transcription factor 6
BIK: BCL2-interacting killer
BIM: BCL-2-interacting mediator of cell death
BRAF: B-Raf proto-oncogene serine/threonine kinase
CHOP: C/EBP homologous protein
DMBC: Department of Molecular Biology of Cancer
ER: Endoplasmic reticulum
ERAD: Endoplasmic reticulum-associated protein degradation
ERK1/2: Extracellular signal-regulated kinase 1/2
GEL: Geldanamycin
GRP78/BiP: Glucose-regulated protein 78/binding immunoglobulin protein
HSP70: Heat shock protein 70
HSP90: Heat shock protein 90
MEK1/2: Mitogen-activated protein kinase 1/2
IRE1α: Inositol-requiring enzyme 1 alpha
NRAS: Neuroblastoma RAS viral oncogene homolog
NQO1: NAD(P)H:quinone oxidoreductase 1
PARP1: Poly(ADP-ribose) polymerase 1
PERK: Protein kinase R-like endoplasmic reticulum kinase
PLX: Vemurafenib an inhibitor of BRAF^V600E^
TRA: Trametinib an inhibitor of MEK1/2
UPR: Unfolded protein response
XBP1 (XBP1s): X-box binding protein 1 (spliced XBP-1)
