# Supplementary material for: 17-Aminogeldanamycin selectively diminishes IRE1α-XBP1s pathway activity and cooperatively induces apoptosis with MEK1/2 and BRAFV600E inhibitors in melanoma cells of different genetic subtypes
Source: Apoptosis. 2019 Apr 15;24(7):596–611. doi: 10.1007/s10495-019-01542-y (PMC6598962; doi:10.1007/s10495-019-01542-y)

**Electronic Supplemental Materials**

**Online Resource 1** Primer sequences, forward (F) and reverse (R) used in the qRT-PCT experiments.

| **Transcript** | **Sequence** |
| --- | --- |
| **BIK** | F: TCTTGATGGAGACCCTCCTGT  R: CAAGAACCTCCATGGTCGGG |
| **BIM** | F: AGTGGGTATTTCTCTTTTGACACAG  R: GTCTCCAATACGCCGCAACT |
| **CHOP** | F: AAGGCACTGAGCGTATCATGT  R: TGAAGATACACTTCCTTCTTGAACAC |
| **GRP78** | F: TTGCGCTGTGCTCCTGTGCT  R: TTCTTGAACACGCCGACGCAGG |
| **HSP70** | F: CGACCTGAACAAGAGCATCA  R: AAGATCTGCGTCTGCTTGGT |
| **RPS17** | F: AATCTCCTGATCCAAGGCTG  R: CAAGATAGCAGGTTATGTCACG |
| **XBP1s** | F: CTGAGTCCGAATCAGGTGCAG  R: GTCCATGGGAAGATGTTCTGG |

|  | **BRAF^V600E^** | | | | **NRAS^Q61R^** |
| --- | --- | --- | --- | --- | --- |
|  | **DMBC12** | **DMBC21** | **DMBC28** | **DMBC29** | **DMBC22** |
| *ATF4* | Q22P +/+  benign 0.001 |  | Q22P +/-  benign 0.001 | Q22P +/-  benign 0.001 | Q22P +/+  benign 0.001 |
| *ATF6* |  | M67V +/+  benign 0.049 | M67V +/+  benign 0.049 | M67V +/+  benign 0.049 | M67V +/+  benign 0.049 |
| *DDIT3* (CHOP) |  |  |  |  |  |
| *EIF2AK3* (PERK) | A704S +/+  benign 0.003  Q166R +/+  benign 0.000  L21del +/+  inframe deletion | A704S +/-  benign 0.003  Q166R +/-  benign 0.000  S136C +/-  benign 0.002  L21del +/-  inframe deletion | A704S +/-  benign 0.003  Q166R +/-  benign 0.000  S136C +/-  benign 0.002  L21del +/-  inframe deletion | A704S +/-  benign 0.003  Q166R +/-  benign 0.000  S136C +/-  benign 0.002  L21del +/-  inframe deletion | Q166R +/+  benign 0.000  S136C +/+  benign 0.002  L21del +/+  inframe deletion |
| *EIF2S1*  (eIF2α) |  |  |  |  |  |
| *ERN1*  (IRE-1α) |  |  |  |  |  |
| *HSPA5* (GRP78) |  |  |  |  |  |
| *XBP1* |  | A7T +/+  benign 0.010 | A7T +/+  benign 0.010 | A7T +/+  benign 0.010 |  |

**Online Resource 2** Mutation status of genes encoding proteins involved in unfolded protein response. Only non-synonymous mutations and indels are included. Mutations are marked as homozygous (+/+) or heterozygous
(+/-). Predictions of functional effects of amino acid substitutions were assessed by Polyphen-2 software. Polyphen-2 predictions were classified based on the Polyphen-2 scores as benign (scores 0.000-0.449). Names of proteins are given in the brackets if they differ from gene names.

**Online Resource 3** Representative microphotographs of melanoma cell lines incubated with either AG or GEL at indicated concentrations for 45 hours. Microphotographs were taken using time-lapse imaging system IncuCyte ZOOM. Green fluorescence relates to active caspase-3/7. Scale bar, 100 μm.


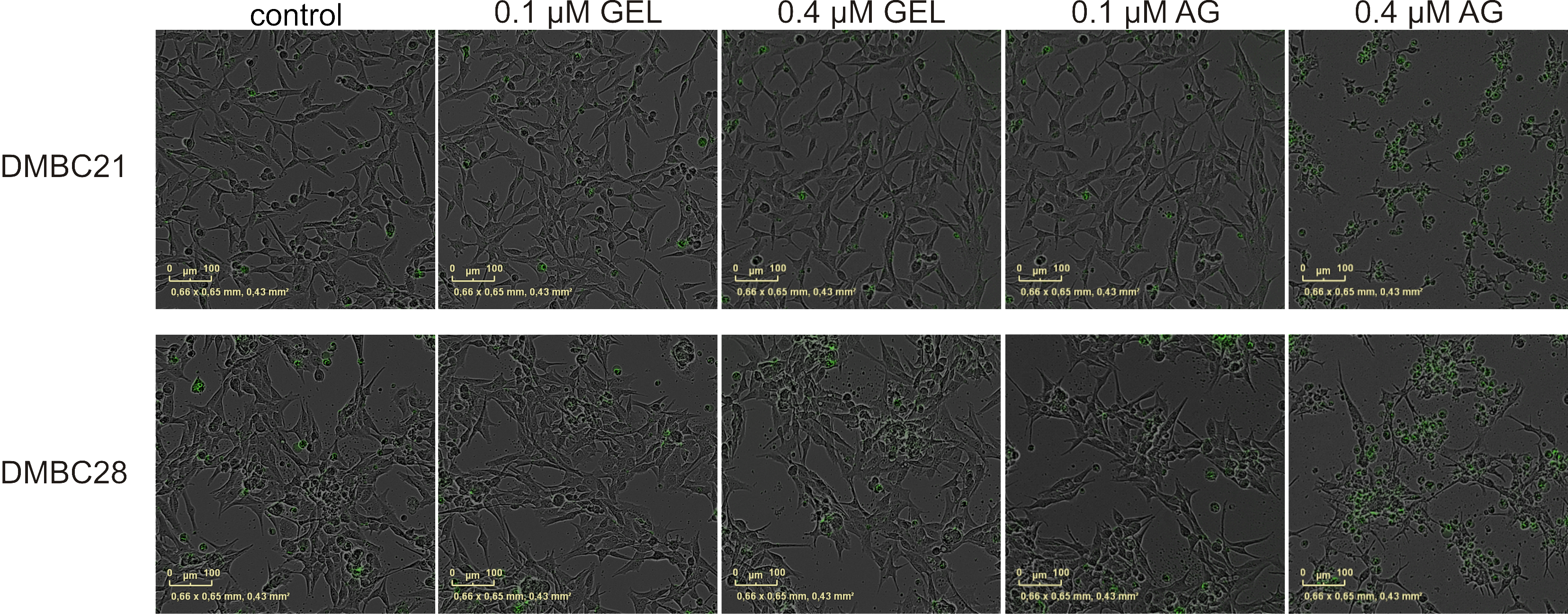


**Online Resource 4** mRNA level of XBP1s was assessed by qRT-PCR after 22 h of cell incubation with 0.1 μM or 0.4 μM GEL, and expressed relatively to the control.


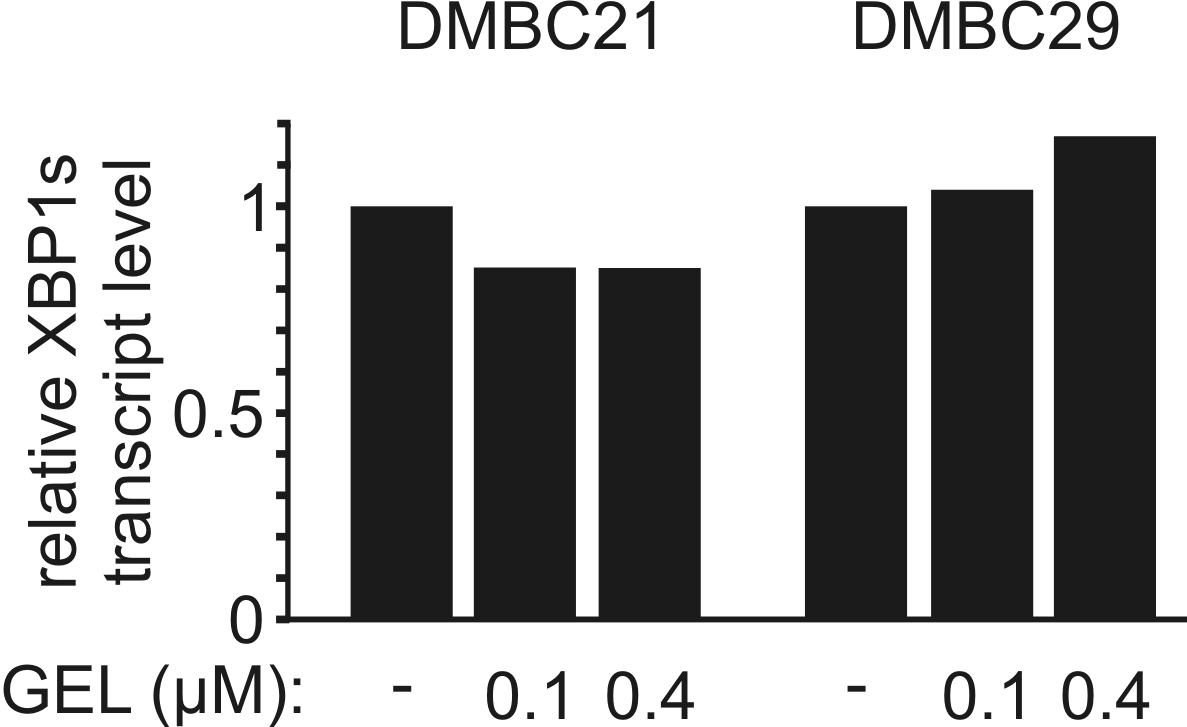


**Online Resource 5** **a** p53 protein level was assessed by Western blotting after 4 and 24 hours of cell incubation with 0.4 μM AG. An equal loading was confirmed by β-actin. Quantification of the protein level is shown below the blots. **b** The transcript level of BIK was assessed by qRT-PCR after 22 h and shown relatively to the control.


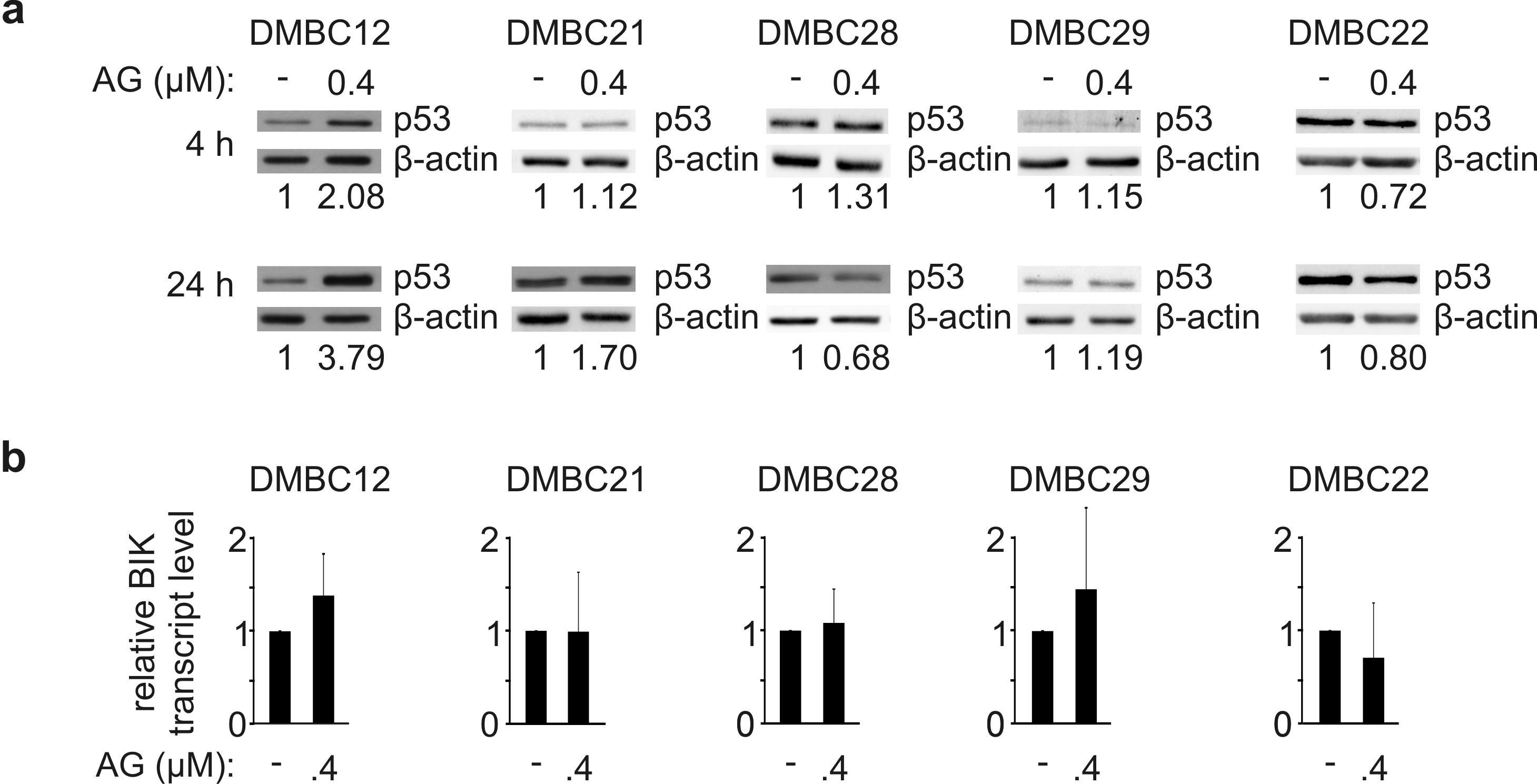


**Online Resource 6** **a-b** mRNA levels of HSP70 and GRP78 were assessed by qRT-PCR after 6 and 22 h of cell incubation with 0.1 μM AG, and expressed relatively to the control. **p*≤0.05; ns, not significant


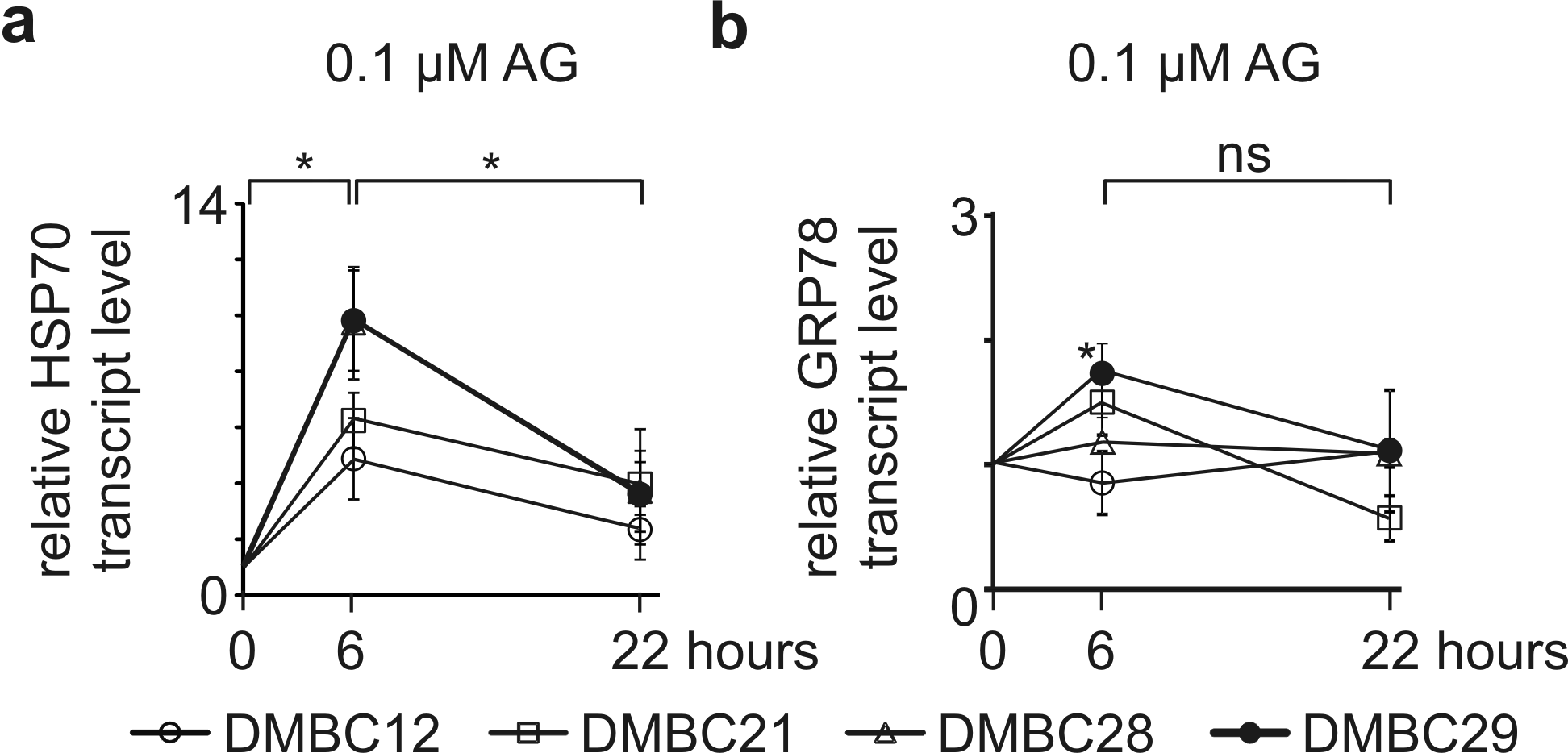


**Online Resource 7** **a** Quantification of cPARP level after 24 hours of incubation with drugs. **b** Level of phosphorylated IRE1α (p-IRE1α) was determined after 24 h of incubation with drugs. β-actin was used as a loading control. XBP1s transcript level was assessed by qRT-PCR after 22 h, and shown relatively to the control.


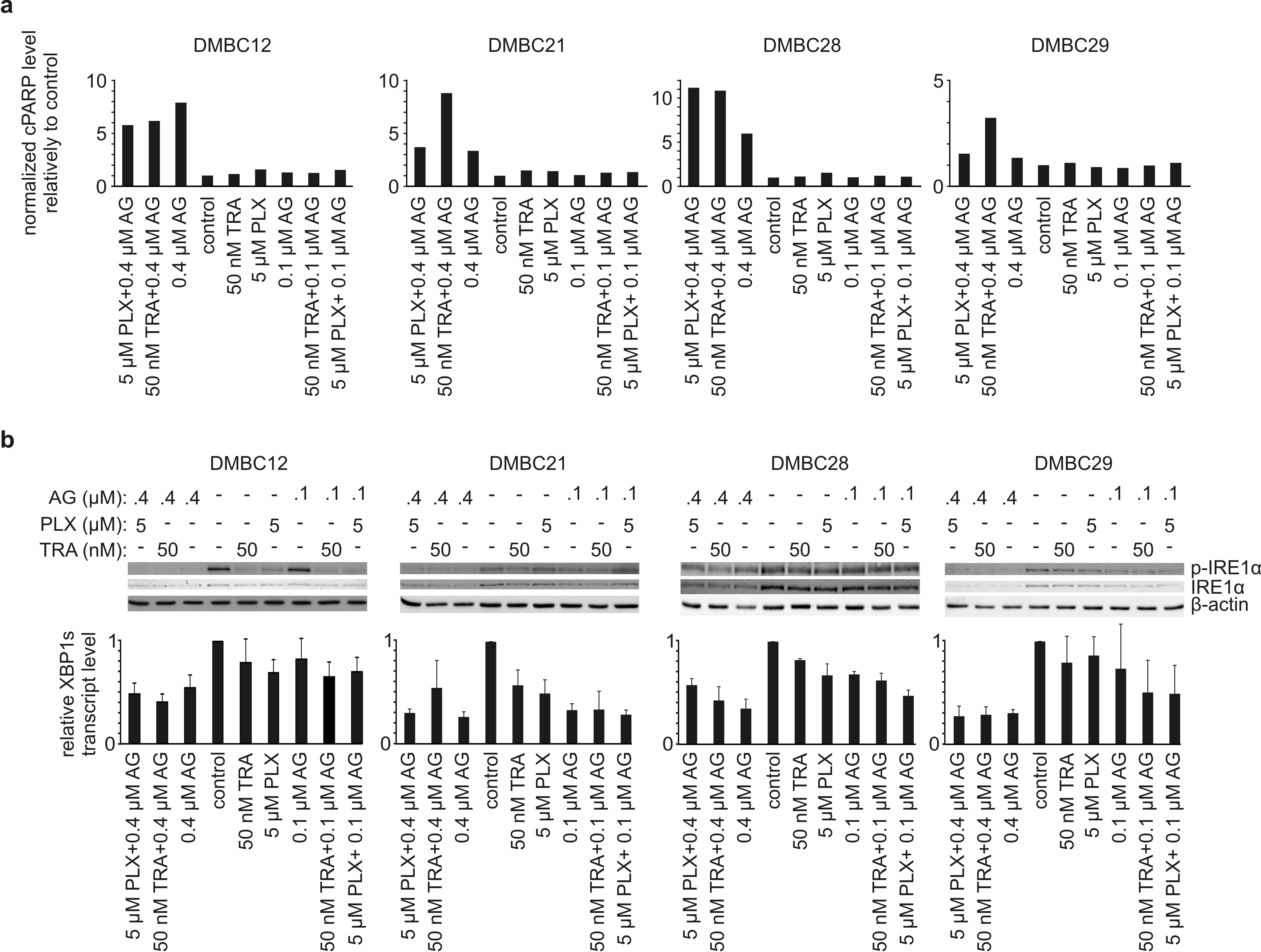

Supplement: Supplementary file 1 — Supplementary material 1 (DOC 6160 kb) [file 10495_2019_1542_MOESM1_ESM.doc]
